# A Stable Chemokine Gradient Controls Directional Persistence of Migrating Dendritic Cells

**DOI:** 10.3389/fcell.2022.943041

**Published:** 2022-08-09

**Authors:** Thomas Quast, Karolin Zölzer, Donald Guu, Luis Alvarez, Carsten Küsters, Eva Kiermaier, U. Benjamin Kaupp, Waldemar Kolanus

**Affiliations:** ^1^ Molecular Immunology and Cell Biology, Life and Medical Sciences Institute (LIMES), University of Bonn, Bonn, Germany; ^2^ Molecular Sensory Systems, Max Planck Institute for Neurobiology of Behavior—Caesar, Bonn, Germany; ^3^ Immune and Tumor Biology, Life and Medical Sciences Institute (LIMES), University of Bonn, Bonn, Germany

**Keywords:** leukocytes, dendritic cells, chemotaxis, gradient, directional sensing

## Abstract

Navigation of dendritic cells (DCs) from the site of infection to lymphoid organs is guided by concentration gradients of CCR7 ligands. How cells interpret chemokine gradients and how they couple directional sensing to polarization and persistent chemotaxis has remained largely elusive. Previous experimental systems were limited in the ability to control fast *de novo* formation of the final gradient slope, long-lasting stability of the gradient and to expose cells to dynamic stimulation. Here, we used a combination of microfluidics and quantitative *in vitro* live cell imaging to elucidate the chemotactic sensing strategy of DCs. The microfluidic approach allows us to generate soluble gradients with high spatio-temporal precision and to analyze actin dynamics, cell polarization, and persistent directional migration in both static and dynamic environments. We demonstrate that directional persistence of DC migration requires steady-state characteristics of the soluble gradient instead of temporally rising CCL19 concentration, implying that spatial sensing mechanisms control chemotaxis of DCs. Kymograph analysis of actin dynamics revealed that the presence of the CCL19 gradient is essential to stabilize leading edge protrusions in DCs and to determine directionality, since both cytoskeletal polarization and persistent chemotaxis are abrogated in the range of seconds when steady-state gradients are perturbed. In contrast to *Dictyostelium* amoeba, DCs are unable to decode oscillatory stimulation of soluble chemokine traveling waves into a directional response toward the wave source. These findings are consistent with the notion that DCs do not employ adaptive temporal sensing strategies that discriminate temporally increasing and decreasing chemoattractant concentrations in our setting. Taken together, in our experimental system DCs do not depend on increasing absolute chemokine concentration over time to induce persistent migration and do not integrate oscillatory stimulation. The observed capability of DCs to migrate with high directional persistence in stable gradients but not when subjected to periodic temporal cues, identifies spatial sensing as a key requirement for persistent chemotaxis of DCs.

## Introduction

Directed cell migration along chemokine gradients is considered essential for embryogenesis, wound healing, cancer metastasis, and immune surveillance. DCs are potent antigen-presenting cells and their capacity to induce adaptive immune response requires their navigation from the site of infection to secondary lymphoid tissues, where they prime naïve T lymphocytes (Steinman et al., 1997; Banchereau et al., 2000; Alvarez et al., 2008; Worbs et al., 2017). In addition to interstitial migration this route includes DC navigation through confined areas, such as the entry into initial afferent lymphatics (Pflicke and Sixt, 2009; Tal et al., 2011; Weber et al., 2013) and the traverse of subcapsular and cortical sinuses to enter the paracortical cords (Braun et al., 2011; Ulvmar et al., 2014). Gradient-guided chemotaxis of DCs therefore allows navigation through complex environments and engagement of rare cell populations, which would otherwise not meet by chance, e.g., DC-T cell interactions. Efficient guidance of motile DCs mainly depends on signaling of the G protein-coupled receptor CCR7, which is upregulated on DCs following pathogen encounter and recognizes the two ligands CCL19 and CCL21 (Förster et al., 1999; Gunn et al., 1999; MartIn-Fontecha et al., 2003; Ohl et al., 2004; Kiermaier et al., 2016; Laufer and Legler, 2018). In a physiological milieu, gradients are presumably not stable over long distances for long periods of time as a result of rapid decay of concentration as a function of distance from the secreting source, hindered chemokine diffusion, chemokine immobilization, or dynamic chemokine release (Weber et al., 2013; Majumdar et al., 2014; Ulvmar et al., 2014; Sarris and Sixt, 2015; Vaahtomeri et al., 2017). Navigation in such complex and dynamic environments is poorly understood. However, it is indisputed that it requires an integration of spatial and temporal cues which allows cells to maintain their direction when the guidance cue fluctuates. Two distinct strategies are proposed for cellular gradient sensing (Dusenbery, 1992). Those cells employing spatial sensing strategies simultaneously compare the chemoattractant receptor occupancy at different positions in the cell (e.g., at the front and rear of a polarized cell), which allows the cell to orient itself towards the gradient direction (Parent and Devreotes, 1999). Those cells employing temporal, or sequential, sensing strategies compare receptor occupancy at successive time points, between which the cell moves from one location to another, and accordingly adjust the bias of direction during locomotion (Macnab and Koshland, 1972; Dusenbery, 1998; Kashikar et al., 2012; Alvarez et al., 2014). Motile cells (e.g., bacteria, sperm) that move at large speed compared to their body size employ temporal sensing strategies. In contrast, chemotaxis of large, much slower, amoeboid cells is typically considered to rely on spatial sensing (Berg and Purcell, 1977; Parent and Devreotes, 1999; Iijima et al., 2002). However, recent studies with *Dictyostelium* amoeba suggest that these cells employ both sensing modalities and that an interplay between cellular memory and adaptive temporal sensing allows cells to maintain their direction when the guidance cue fluctuates (Huang and Iglesias, 2014; Nakajima et al., 2014; Skoge et al., 2014). Furthermore, it was recently proposed for myeloid cells that rising chemokine concentrations are required to promote long-range directional migration, implying that temporal sensing controls prolonged responses to chemotactic cues (Petrie Aronin et al., 2017).

Therefore, the existing literature is inconsistent about how chemotactic cues regulate directional locomotion of leukocytes and we still lack a quantitative understanding of how cells interpret both stable and dynamic guidance cues in complex environments. Previous assays such as point release of chemokines from micropipettes (Weiner et al., 1999), under agarose assays (Wilkinson et al., 1983), Dunn chemotaxis chambers (Zicha et al., 1991), and diffusion-based microfluidic approaches in 3D matrices (Haessler et al., 2011; Petrie Aronin et al., 2017; Frick et al., 2018) were limited in the ability to control the shape of the gradient with high spatio-temporal precision and to expose cells to controlled dynamic stimulation. Here, we utilize a combination of a flow-based microfluidic approach and quantitative confocal live cell imaging which allows to directly visualize and track cells during migration and to analyze their response in complex, precisely controlled chemokine fields.

In this study we employ chemotaxis of dendritic cells (DCs) to explore quantitative aspects of directional gradient sensing in a prototypical example of fast migrating leukocytes, which share many characteristics of so-called amoeboid crawling movement described for *Dictyostelium discoideum* (Friedl et al., 2001; Lämmermann and Sixt, 2009; Ishikawa-Ankerhold et al., 2022). We provide evidence that guidance control of DCs strongly depends on the integration of spatial information, since cells depolarize and stop in the range of seconds following complete CCL19-gradient abrogation, respectively. The observed capability of DCs to migrate with high directional persistence in static gradients but not in temporally dynamic environments strongly argues for a spatial navigation strategy.

## Materials and Methods

### Mice

All mice used in this study were bred on a C57BL/6J background, maintained at the institutional animal facility, and sacrificed at 7–10 weeks of age for isolation of organ (bone, spleen) according to the German law of animal experimentation (German Protection of Animals Act, Deutsches Tierschutzgesetz § 11; § 4, Satz 3). Lifeact-EGFP mice (Riedl et al., 2010) were a gift from Frank Bradke (DZNE, Bonn).

### Generation, Cell Culture and Maturation of Bone Marrow Derived Dendritic Cells

Dendritic cells (BM-DCs) were generated from the bone marrow extracted from femur and tibia of 7–10-week-old mice as described previously (Quast et al., 2011). We employed DCs from Lifeact-EGFP mice, which allow visualization of F-actin without interfering with polymerization dynamics (Riedl et al., 2010). In brief, bone marrow cells were collected by flushing the bones with PBS (without Ca^2+^, Mg^2+^; Pan Biotech). Subsequently, 5 × 10^6^ bone marrow cells were cultured in 10 cm petri dishes (Greiner Bio-one) in 10 ml complete cell culture medium [VLE-RPMI 1640 (Pan Biotech), supplemented with 10% Fetal Calf Serum (FCS, Sigma-Aldrich, Pan Biotech), 100 µ/ml Penicillin (Pan Biotech), and 100 μg/ml Streptomycin (Pan Biotech)] containing 10 ng/ml recombinant Granulocyte-Macrophage Colony Stimulating Factor (GM-CSF, Peprotech). On day 3, cells were fed by addition of 5 ml complete cell culture medium supplemented with 10 ng/ml GM-CSF. On day 6, half of the cell culture medium in stock was replaced by fresh complete cell culture medium supplemented with 10 ng/ml GM-CSF. To induce maturation, BM-DCs were stimulated overnight with 200 ng/ml Lipopolysaccharide (LPS) from *E. coli* O127:B8 (Sigma-Aldrich) and used for experiments on day 7–9.

### Purification of Splenic Dendritic Cells

Dendritic cells were purified from spleens of 7–10-week-old Lifeact-EGFP mice (Riedl et al., 2010). Collagenase solution, consisting of 0.2 mg/ml collagenase IV (Sigma-Aldrich), 10% FCS, 100 µ/ml DNAse I (Invitrogen) in HBSS, was added to the spleens after they were minced into small pieces and incubated at 37°C for 45–60 min. The dissociated tissue was homogenized using syringe and 19G cannula and subsequently sieved through cell strainer. The cell suspension was washed once with PBS containing 5% FCS and incubated with a so-called ACK (Ammonium-Chloride-Potassium) lysis buffer at room temperature for 5 min. The ACK lysis buffer is used for the lysis of red blood cells and consists of 155 mM NH_4_Cl, 10 mM KHCO_3_, 0.1 mM EDTA in ddH_2_O. Lysis was stopped with excess of PBS followed by washing twice with PBS. DCs were purified from cell suspension using CD11c Microbeads Ultrapure and the autoMACS Pro Separator (both from MiltenyiBiotec). CD11c+ DC were subsequently cultured in six well plates (Greiner Bio-one) overnight in 5 ml complete cell culture medium [VLE-RPMI 1640 (Pan Biotech), supplemented with 10% Fetal Calf Serum (FCS, Sigma-Aldrich, Pan Biotech), 100 µ/ml Penicillin (Pan Biotech), and 100 μg/ml Streptomycin (Pan Biotech)] containing 10 ng/ml recombinant Granulocyte-Macrophage Colony Stimulating Factor (GM-CSF, Peprotech). To induce maturation, BM-DCs were stimulated for 6 h with 200 ng/ml Lipopolysaccharide (LPS) from *E. coli* O127:B8 (Sigma-Aldrich) and subsequently used for experiments.

### Cell Culture of *Dictyostelium* Amoeba and Preparation

Cells of *D. discoideum* strain AX2 (clone: EB27-3-4), expressing LimEdeltacc-GFP as an actin probe, were a gift from Günther Gerisch (MPI of Biochemistry, Martinsried). The cells were cultivated at 22°C in 10 cm cell culture dishes (Greiner Bio-one) with nutrient medium containing 10 μg/ml of G418 (Sigma-Aldrich). Nutrient medium consists of 7.15 g Bacto™ Yeast Extract (Thermo Fisher), 14.3 g Bacto™ Peptone (Thermo Fisher), 18.0 g D-(+)-maltose monohydrate (Sigma-Aldrich), 0.0486 g KH_2_PO_4_ (Roth), 0.616 g Na_2_HPO_4_ * 2H_2_O (Roth) in 1 L cell culture water (Sigma-Aldrich), was adjusted to pH 6.7, and subsequently filtered by the use of a 0.45 µm porous membrane (Filtropur; Sarstedt). The cells were split every 2–3 days in a ratio of 1:5–1:10, before the cell monolayers became confluent. For starvation, 5 * 10^6^ cells were shaken overnight (60 turns/min) at 22°C in an Erlenmeyer flask (volume: 10 ml) with 3 ml phosphate buffer (17 mM; 2.0 g KH_2_PO_4_ and 0.356 g Na_2_HPO_4_ * 2H_2_O in 1 L cell culture water, pH 6.0) placed on a shaker with orbital motion (GFL 3015; Fisher Scientific) and subsequently used for microfluidic experiments.

### Chemoattractants and Gradient Visualization

Recombinant murine chemokines (CCL19, CXCL12; Peprotech) were reconstituted to a concentration of 100 μg/ml in cell culture water and stored at −20°C. Prior to use, the chemokine was diluted to a working concentration of 50 nM in so-called Microfluidic buffer, i.e., Hank’s balanced salt solution (HBSS, with Ca^2+^, Mg^2+^; Pan Biotech), supplemented with 0.5% FCS (Sigma-Aldrich, Pan Biotech) and 25 mM HEPES buffering agent (Pan Biotech). Alexa Fluor™ 647 dextran, 10,000 MW (Thermo Fisher Scientific) was prepared following manufacturer’s protocol and reconstituted to a concentration of 1 mM in PBS and stored at −20°C. Prior to use, Alexa Fluor™ 647 dextran was diluted to a working concentration of 300 nM in Microfluidic buffer. The fluorescently labeled dextran is characterized by a similar molecular weight as compared to the recombinant CCL19 (or CXCL12) and served as a surrogate molecule to monitor the chemokine gradient within the microfluidic chamber. The synthetic peptide WKYMVM (Biotechne, Tocris) is a selective agonist for the formyl peptide receptors FPR2 and FPR3. WKYMVM was reconstituted to a concentration of 100 µM in cell culture water and stored at −20°C. Prior to use, WKYMVM was diluted to a working concentration 50 nM in Microfluidic buffer. LTB4 (Sigma-Aldrich) was reconstituted to a concentration of 100 µM in cell culture water and stored at −20°C. Prior to use, LTB4 was diluted to a working concentration 25 nM in Microfluidic buffer. cAMP (A9501, Sigma-Aldrich) was reconstituted to a concentration of 1 mM in cell culture water and stored at −20°C. Prior to use, cAMP was diluted to a working concentration of 1 µM in cell culture water. Alexa Fluor™ 633, (Thermo Fisher Scientific) was prepared following manufacturer’s protocol and reconstituted to a concentration of 1 mM in PBS and stored at −20°C. Prior to use, Alexa Fluor™ 633 was diluted to a working concentration of 300 nM in PBS and served as a surrogate molecule to monitor gradients of cAMP, LTB4, or WKYMVM, respectively.

### Coating of Microfluidic Chamber-Slides

Chamber-slides (µ-slide 3-in-1, ibiTreat; Ibidi) were coated with fibronectin to allow integrin-mediated adherence and migration of BM-DCs and CD11c+ DCs. Fibronectin from human plasma (Sigma-Aldrich) was reconstituted to a concentration of 200 μg/ml in cell culture water and stored at −20°C. Prior to use, fibronectin was diluted to a working concentration of 50 μg/ml in PBS and manually filled into the chamber-slide through the outlet adapter. The chamber-slide was incubated with fibronectin for 1 h at room temperature and subsequently washed with PBS. Then the coated chamber-slide was blocked with Casein to completely inhibit unwanted immobilization of soluble chemokines. Casein (Hammerstein grade; MP Biomedicals) was reconstituted to a working concentration of 2.5 vol.-% in PBS and stored at −80°C; incubation was performed for 1 h at room temperature followed by washing with Microfluidic buffer, supplemented with 0.5% FCS (Sigma-Aldrich) and 25 mM HEPES buffering agent (Pan Biotech).

### Seeding of Bone Marrow-Dendritic Cells in Microfluidic Chamber-Slides

For chemotaxis experiments non-adherent LPS-stimulated mature BM-DCs were collected from the cell culture medium supernatant. Then the cells were resuspended in Microfluidic buffer and subsequently placed in a microfluidic chamber-slide in 100 μl at a cell density of 1.5 * 10^6^ cells/ml. Cells were settled for adherence for 60 min at 37°C/5%CO_2_/humidity. Before live cell imaging the channel was washed once with Microfluidic buffer to remove non-adherent cells.

### Seeding of Splenic CD11c+ Dendritic Cells in Microfluidic Chamber-Slides

For chemotaxis experiments CD11c+ DCs were resuspended in Microfluidic buffer and subsequently placed in a microfluidic chamber-slide in 100 μl at a cell density of 1.5 * 10^6^ cells/ml. To improve cell adhesion of CD11c+ DCs, cells were incubated with MgCl_2_ to increase activation of integrins. To this end CD11c+ DCs were resuspended in a modified Microfluidic buffer, consisting of Hank’s balanced salt solution, supplemented with 0.5% FCS (Sigma-Aldrich), and 25 mM HEPES buffering agent (Pan Biotech), and 10 mM MgCl_2_. Cells were settled for adherence for 60 min at 37°C/5%CO_2_/humidity. Before live cell imaging the channel was washed once with Microfluidic buffer to remove non-adherent cells.

### Seeding of *Dictyostelium* Amoeba in Microfluidic Chamber Slides

Following starvation 0.5 * 10^6^ AX2 cells/ml in 17 mM phosphate buffer were placed into uncoated chamber-slides (µ-slide 3-in-1, ibiTreat; Ibidi) and incubated for 20 min at 22°C before chemotaxis experiments were performed with adherent cells.

### Generation of Precise Concentration Gradients Using the Microfluidic Device

**FIGURE 1:**
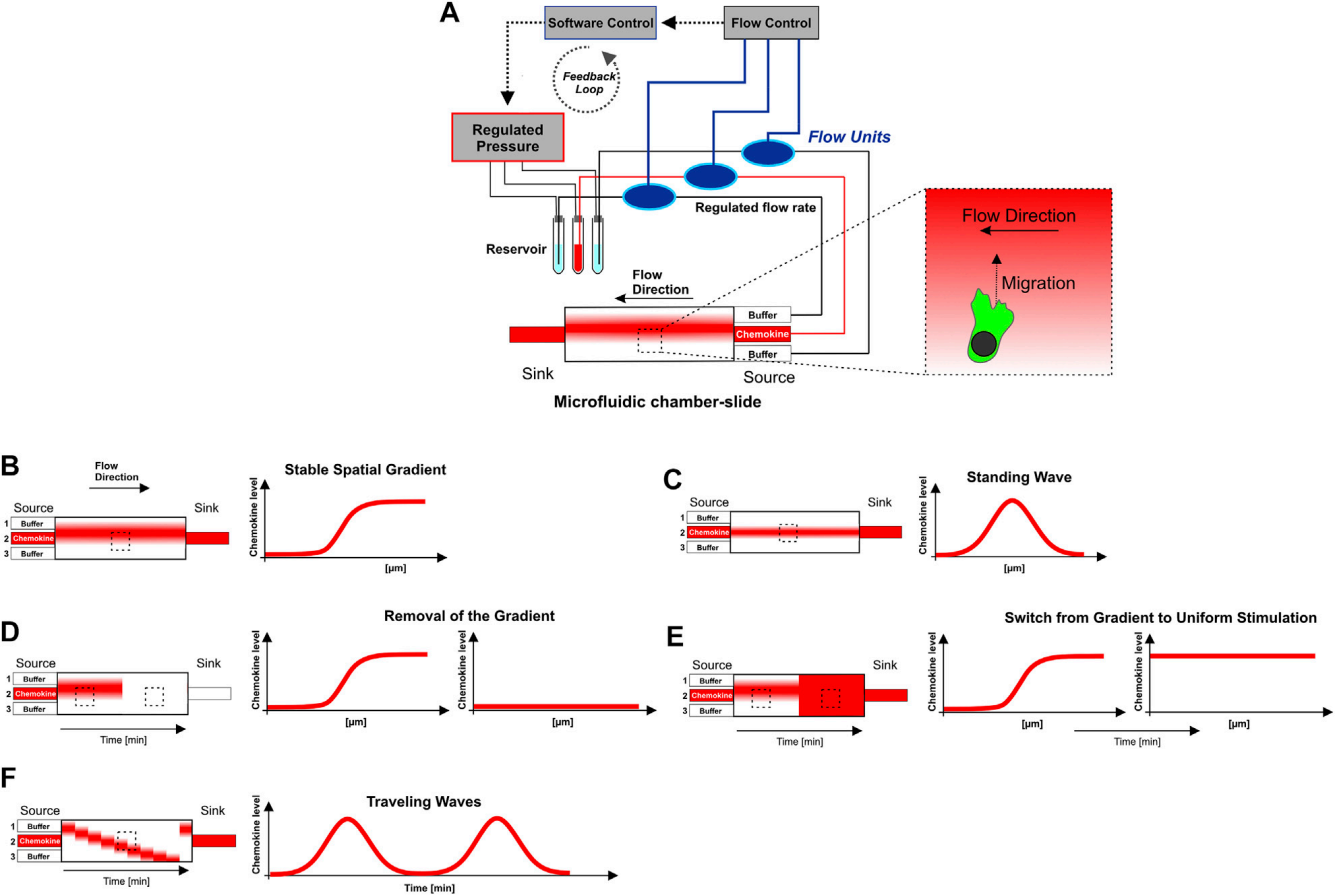
Microfluidic approach to generate sigmoidal shaped concentration gradients with high spatio-temporal precision. **(A)** Graphical representation of the “source-sink”-based microfluidic setting. The core piece of this approach consists of a chamber-slide in which the cells are placed before they are exposed to the gradient. In the “source-sink”-based microfluidic approach, laminar flows from three independent source channels were merged and hydro-dynamically focused to generate soluble concentration gradients of chemokines with a sigmoidal-shaped concentration profile. The three inlet channels (source) are connected via flexible tubes to centrifuge tubes, which are used as a reservoir of the flow buffer source (central inlet: chemokine + fluorescent 10 kDa-Dextran, both outer inlets: flow buffer without chemokine). One more flexible tube is connected to the outlet (sink) and ends in a waste vessel. Pressure is controlled *via* a pneumatic pressure regulator. High precision bidirectional flow sensors interfaced with control software allows for direct feedback control and monitoring of individual flow rates. Pressure control is automated by control software using custom-made scripts that set the flow rates from the individual inlet dynamically over time. **(B–F)** Graphical representation of flow-based soluble steady-state (stable gradient, standing wave) and dynamic chemokine environments (removal of the gradient, switch from gradient to uniform stimulation, and traveling waves). For the generation of dynamic traveling waves modified executable shell scripts from Nakajima and Sawai (2016) are used. The observation region within the microfluidic chamber-slide is indicated by the rectangle with the dashed line. Chemotaxis of cells towards the gradient is perpendicilar to flow direction. The experimental setup is explained in detail in the *Materials and Methods* section. Supplementary Table S1 summarizes flow rates as well as the spatio-temporal condition of the gradient (stable, dynamic) which were used in respective experiments.

To generate spatially and temporally precisely controlled soluble concentration gradients of chemoattractant (CCL19, CXCL12, LTB4, FPR2/3 receptor agonist WKYMVM, cAMP, respectively) by laminar flow, we employed a source-sink-based microfluidic approach. The core piece of this approach consists of a chamber-slide (µ-slide 3-in-1, ibiTreat; Ibidi) that merges three separated liquids into one channel (Figure 1A). The three inlet channels (source) were connected to 50 ml centrifuge tubes (Corning), which were used as a reservoir of the flow buffer source (Microfluidic buffer). Flexible tubes (Idex; Bola) from the buffer source without chemoattractant were connected to both outer inlets of the chamber-slide, whereas a flexible tube from the buffer source with chemoattractant (and Alexa Fluor™ 647 dextran or Alexa Fluor™ 633) was connected to the central inlet as depicted in the graphical representation (Figure 1A). One more flexible tube was connected *via* Luer connector (Ibidi) to the outlet (sink) and ends in a waste vessel. Air-tight connectors (P-Cap; Fluigent) allowed pressurization of Falcon tubes for microfluidic applications. Before capping and connecting the Falcon tubes with the buffer source the solution was purged with gaseous nitrogen for 5 min followed by removal of emergent air bubbles with a cell culture pump. This prevents unwanted gas bubbles, which hinder the formation and maintenance of stable gradients in the chamber. Pressure is precisely controlled *via* a pneumatic pressure regulator (MFCS™-EZ; Fluigent). High precision bidirectional flow sensors (Flow Unit; Fluigent) interfaced with *Maesflow* software (Fluigent) allows for direct feedback control and monitoring of individual flow rates. Pressure control was automated by the *Maesflow* software (Fluigent) using custom-made scripts that set the flow rates from the individual inlet dynamically over time. For the generation of dynamic traveling waves we modified executable MFCS shell scripts named “travelingwave.sh.” from Nakajima and Sawai (2016). Supplementary Table S1 summarizes flow rates as well as the spatio-temporal condition of the gradient (stable, dynamic) which were used in respective experiments.

### Confocal Live Cell Imaging

**FIGURE 2:**
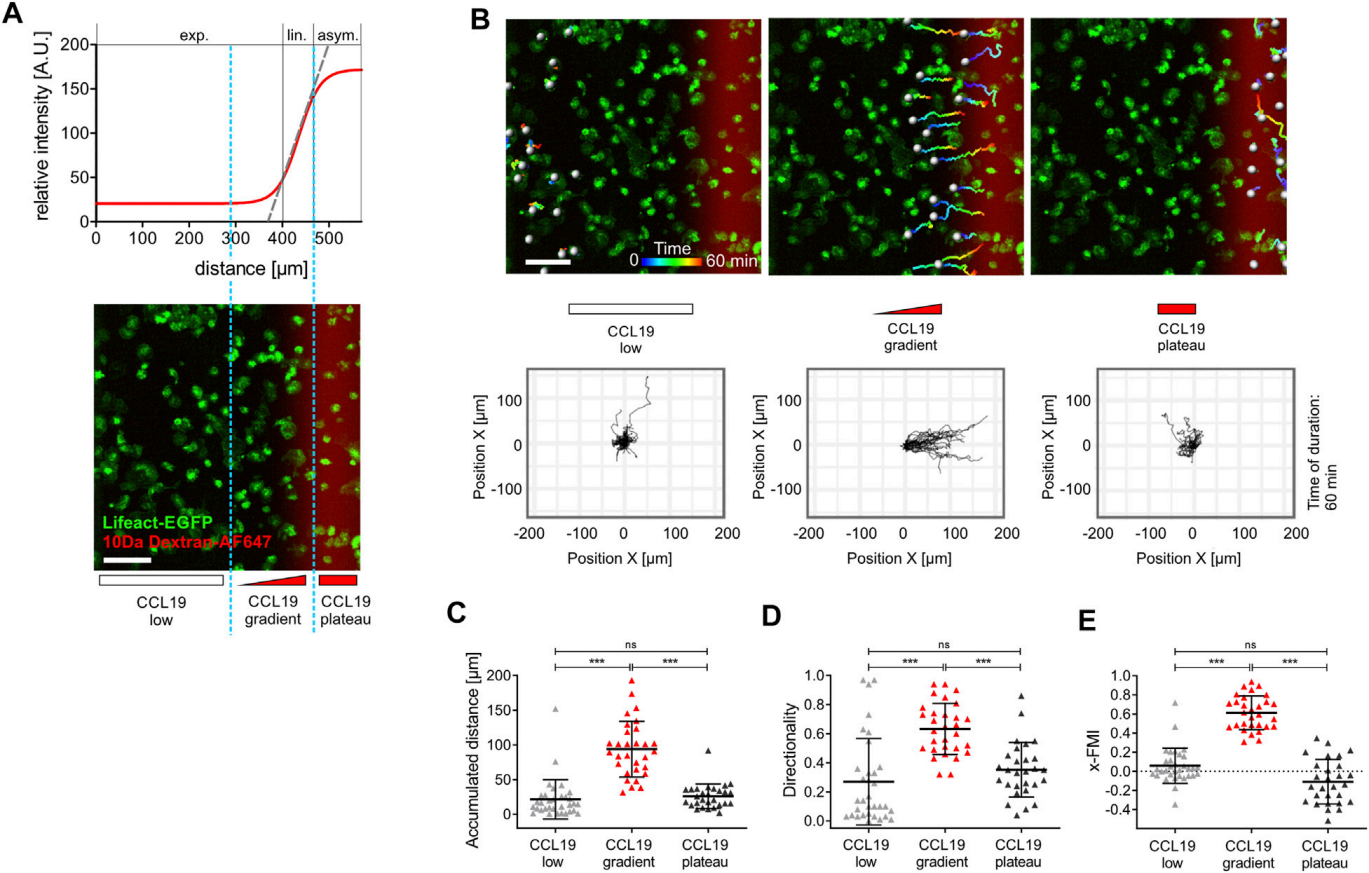
Spatial sensing and chemotactic response of bone marrow-derived dendritic cells (BM-DCs) in stable soluble CCL19 gradients. **(A)** Sigmoidal shaped concentration profile of a stable soluble gradient analyzed with a surrogate molecule, 10 kDa Dextran Alexa Fluor (AF) 647; exp., exponential; lin., linear; asym., asymptotic. Light blue dashed line indicates zoning in three areas with different gradient steepness for separated cell tracking analyses: “CCL19 low,” “CCL19 gradient,” “CCL19 plateau” (upper and lower panel). Scale bar = 100 µm. **(B)** Pseudo-colored tracks of individual motile Lifeact-EGFP expressing BM-DCs (time is color-coded: blue = 0 min, red = 60 min) following cell tracking analysis in “CCL19 low,” “CCL19 gradient,” “CCL19 plateau”-areas, respectively. Green = Lifeact-EGFP expressing BM-DCs, red = 10 kDa Dextran AF 647 (upper panel). Corresponding plots of cell trajectories (lower panel). Scale bar = 100 µm. Tracks of individual areas are representative of five experiments with 28–33 cell tracks per area. **(C–E)** Analysis of BM-DCs migration for 60 min, including **(C)** accumulated distance, **(D)** directionality, **(E)** x-Forward Migration Index (x-FMI) of individual cells. Representative data from one of five experiments; triangles represent randomly chosen cells per area, *N* = 28–33, mean ± SD. Statistical tests: **(C–D)** ***p ≤ 0.001, ns, non-significant, Dunn’s multiple comparison (post-hoc Kruskal-Wallis test). See also Supplementary Figure S1 and Supplementary Video S1.

**FIGURE 3:**
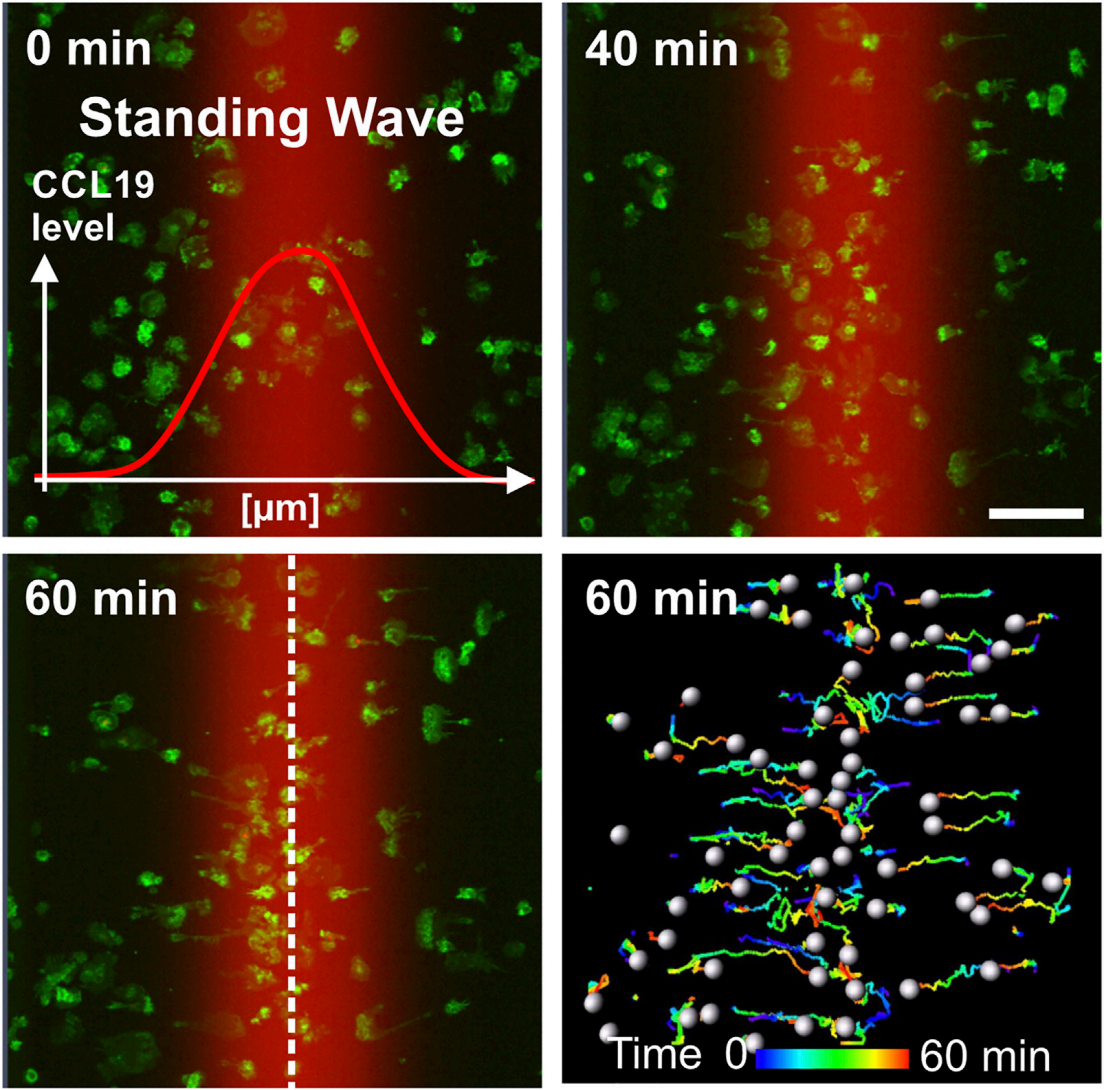
Directional migration of BM-DCs towards single standing CCL19 waves. Analysis of BM-DCs migration towards both halves of a symmetric bell-shaped standing CCL19 wave. Tracks of individual motile Lifeact-EGFP expressing BM-DCs are pseudo-colored (time is color-coded: blue = 0 min, red = 60 min). Peak region of the standing wave is indicated by white dashed line. Scale bar = 100 µm. See also Supplementary Video S2.

**FIGURE 4:**
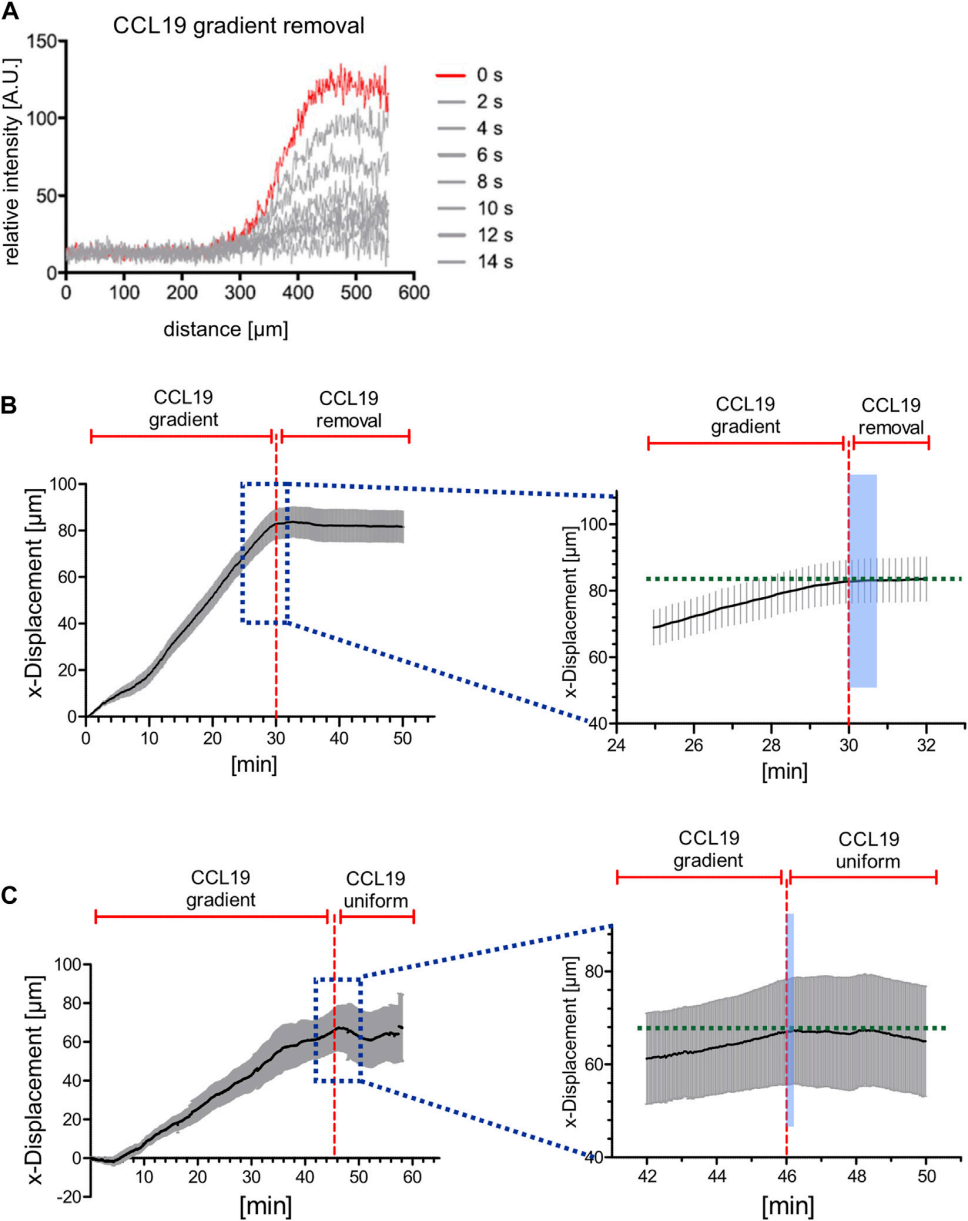
Directional persistent migration of BM-DCs is abrogated in the range of seconds when stable CCL19 gradients are perturbed. **(A)** Concentration profiles of soluble 10 kDa Dextran Alexa Fluor (AF) 647, which is used as a surrogate molecule to mirror CCL19 concentration profiles, before (red line, *t* = 0 s) and after (gray lines) flow-based gradient removal. **(B)** x-displacements of motile BM-DCs towards a stable soluble CCL19 gradient and following CCL19 gradient removal (instant time of gradient abrogation indicated by red dashed line). Stopping time of chemotaxing cells was quantified by analyzing the stagnation of x-displacement in response to gradient removal (indicated by the light blue area in the right panel, which is an enlarged viewing of the dark blue dotted area in the left panel). **(C)** x-displacements of motile BM-DCs towards a stable soluble CCL19 gradient and following uniform CCL19 stimulation (instant time of gradient abrogation indicated by red dashed line). Stopping time of chemotaxing cells was quantified by analyzing the stagnation of x-displacement in response to gradient removal (indicated by the light blue area in the right panel, which is an enlarged viewing of the dark blue dotted area in the left panel). **(A,B)** Representative data from one of three experiments, *N* = 12–15, mean ± SEM. See also Supplementary Videos S3, S4.

**FIGURE 5:**
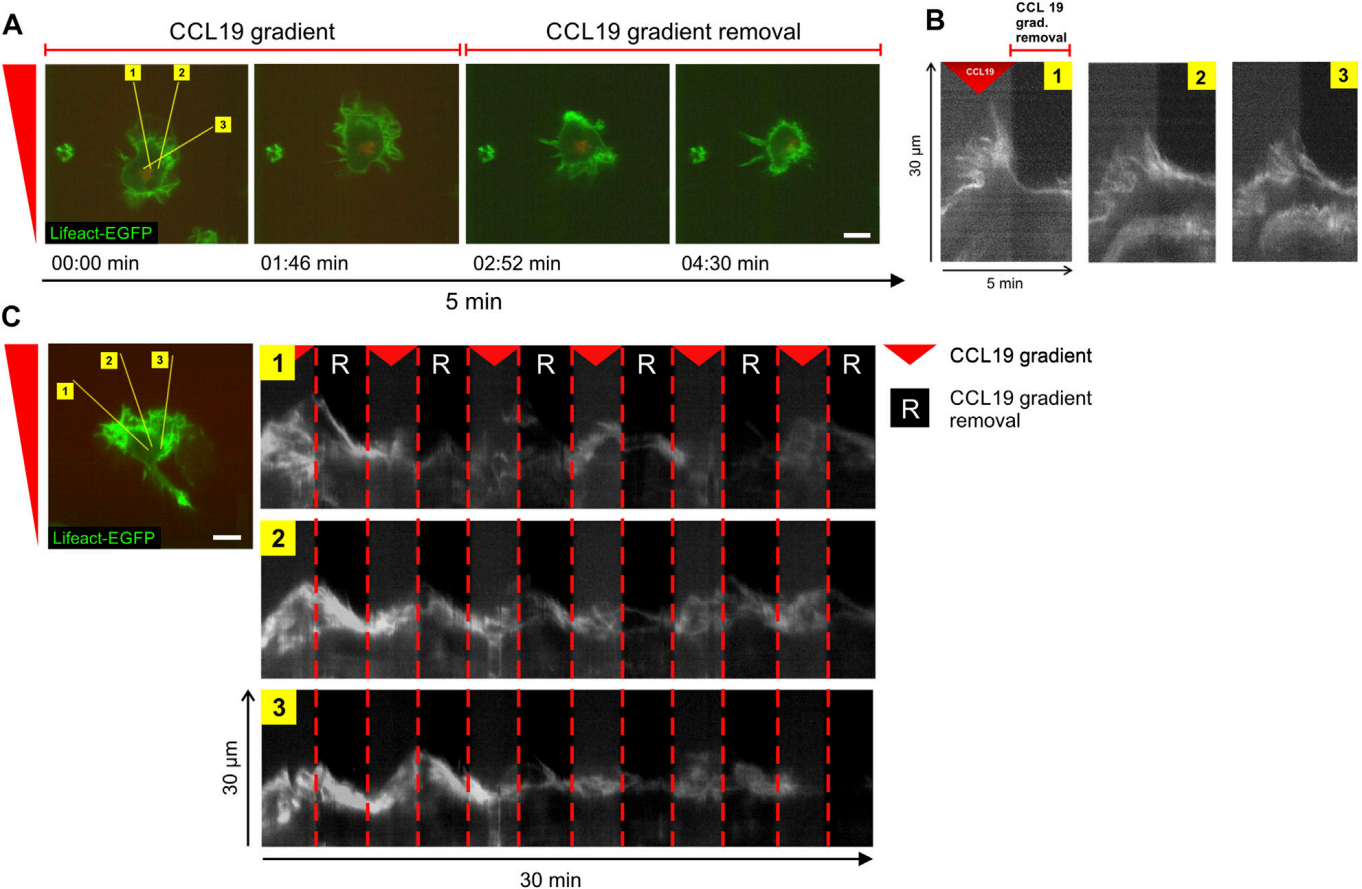
Steady-state chemokine-gradients are essential to stabilize leading edge protrusions in BM-DCs. **(A,B)** Time-lapse confocal imaging sequence of actin dynamics and leading-edge polarization of a single cell in a stable soluble CCL19 gradient (for 2.5 min) and following CCL19 gradient removal (for 2.5 min). Lifeact-EGFP expressing BM-DC was visualized over a period of 5 min at 2 s per frame. The yellow lines in **(A)** mark kymographs (time-space-plots) plotted in **(B)**. The kymographs display actin dynamics of the regions over time. **(C)** Time-lapse confocal imaging sequence of actin dynamics and leading-edge polarization of a single cell exposed to sequential removal and re-apply of stable soluble CCL19 gradients. Lifeact-expressing BM-DC was visualized over a period of 30 min at 2 s per frame. The yellow lines (left panel) mark kymographs (time-space-plots) plotted on the right panel. Green = Lifeact-EGFP. Red triangle mark presence and “R” absence of stable soluble CCL19 gradients. Scale bars = 10 µm. Representative data from one of five experiments. See also Supplementary Video S5.

**FIGURE 6:**
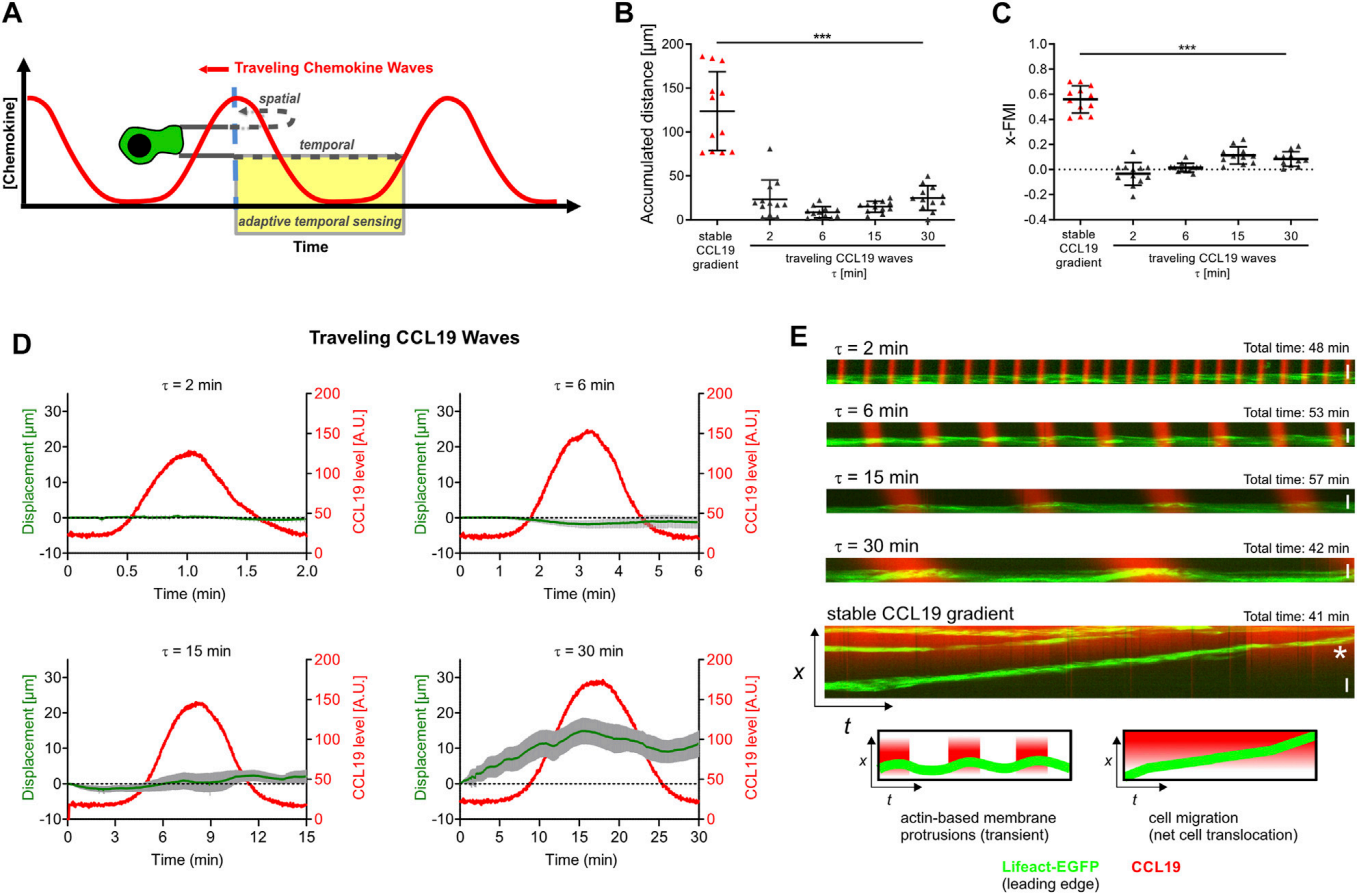
BM-DCs are not able to decode oscillatory stimulation of soluble CCL19 traveling waves into a directional chemotactic response. **(A)** Proposed model of spatial and temporal sensing behavior in traveling waves of chemoattractant, modified after (Nakajima et al., 2014; Skoge et al., 2014). **(B,C)** Analysis of BM-DC migration for 60 min exposed to stable soluble CCL19 gradients or traveling CCL19 waves with various periods (τ = 2–30 min), including **(B)** accumulated distance and **(C)** x-Forward Migration Index (x-FMI) of individual cells. Representative data from one of eight experiments; triangles represent randomly chosen cells per condition, *N* = 12, mean ± SD. **(D)** X-displacements of BM-DCs over 60 min migration towards the source of soluble traveling CCL19 waves with various periods (τ = 2–30 min). Representative data from one of eight experiments with randomly chosen cells per condition, *N* = 12, mean ± SEM. **(E)** High resolution kymographic analysis of actin dynamics of Lifeact-EGFP expressing BM-DCs which are exposed to traveling CCL19 waves with various periods (τ = 2–30 min) or soluble stable CCL19 gradients, respectively. Actin dynamics of individual cells was analyzed by kymograph and line scan analysis of yellow lines (indicated in Supplementary Figures S2D–H). White star indicates persistent displacement of the leading edge (EGFP-Lifeact signal), indicating directional net cell translocation. Representative data from one of three experiments for each condition, *N* = 3–12. **(A,E)** Green = Lifeact-EGFP expressing BM-DCs, red = 10 kDa Dextran AF 647. Scale bars = 20 µm **(E)**. See also Supplementary Figures S2D–H and Supplementary Videos S2, S7. Statistical tests: **(B,C)** ***p ≤ 0.001, post-hoc Kruskal-Wallis test.

Image series data of motile DCs were obtained using an inverted confocal ultra-fast laser scanning microscope (5 Live; Zeiss) equipped with a motorized microscope stage (Märzhäuser) and a controllable heating incubation insert (37°C, Heating Work-plate 2000; TempController 2000-2; Pecon). Alternative use of a complex incubation box (37°C, 5% CO_2_) was proved as unsuitable for this microfluidic setup, because the intake airflow caused unwanted air bubbles in the chamber slide and the flexible tubes and subsequently abrogated the stability of the gradient. Image data of motile *Dictyostelium* Amoeba (AX2-cells) were obtained at 22°C. For visualization 488 and 633 nm laser lines and a photomultiplier tube (Zeiss) was used. Objective lens used were ×10 (EC Plan-Neofluar, NA 0.3; Zeiss) for visualization and quantification of chemotaxis (bulk cell analysis, Figures 2–4, 6; Supplementary Figures) and ×63 (Plan-Apochromat, NA 1.40, oil immersion; Zeiss) for visualization of cell polarization and actin dynamics on a single-cell level (Figure 5). Fluorescence images were acquired at one frame per 2–10-s.

### Cell Tracking Analysis

Automated cell tracking was performed with Imaris software (Version 7.6.5; Oxford Instruments). Using green fluorescence of EGFP as a source channel of motile cells we tracked spots over time. The so-called quality filter was set to a level above 5 after spot detection. The Autoregressive Motion Algorithm was used for tracking analysis. A value of 20 µm was set as a maximal distance of individual spots from frame to frame. Image series with annotated pseudo-colored (time-coded) cell tracks were stored as videos (mp4. file format). Cell migration parameters, i.e., accumulated distance (µm), x-displacement (µm), and cell directionality were exported from Imaris software and further analyzed with GraphPad Prism 9.3.1 (GraphPad Software). The x-Forward Migration Index (x-FMI) is similar to a so-called Chemotactic Index. X-FMI was used in this study to calculate directional persistence, i.e., the ratio of the cell’s most direct path to the chemokine gradient source to its accumulated distance. The x-FMI was calculated with a custom-made script using R-software (Version 4.0.3) on the basis of “Position”- and “Track displacement length”- data obtained from Imaris. Stopping time of chemotaxing cells was quantified by analyzing the stagnation of x-displacement in response to gradient removal (Figure 4, indicated by the light blue area in the right panel, which is a magnification of the dark blue dotted area in the left panel).

Manual tracking was performed using the Manual Tracking and Chemotaxis Plugin of Fiji Software (ImageJ 1.53i; NIH, United States) in Figure 4 and Supplementary Videos S3, S4, S6.

### Kymograph Analysis of Actin Dynamics and Membrane Protrusion Formation

Actin dynamics and membrane protrusion formation were analyzed by line-scan and kymograph analysis, using Fiji Software (ImageJ 1.53i; NIH, United States). Image series of the EGFP-channel were processed using the Walking Average plugin to increase signal-to-noise ratio and to flatten the background. Then a segmented line (1 pixel width) which includes the entire leading-edge activity of the cell during the image series was placed from the cell’s center into peripheral cell regions which are exposed to the source direction of the chemoattractant (yellow lines in Figure 5 and Supplementary Figures S2D–H). Subsequently, a kymograph within the range of the segmented line was plotted using the Multiple Kymograph plugin. The resulting kymograph (Time-Space-Plot) represents the gray value changes, i.e., actin dynamics, over time. Superimposition (merging) of EGFP- and Alexa Fluor 647 channels allows correlating the actin-driven protrusion formation (Figures 5, 6E; Supplementary Figures S2D–H).

### Quantification and Statistical Analysis

Reproducibility of the experimental findings was verified using biological replicates, which were performed as independent experiments and designated in the figure legends. Individual experiments were validated separately. Statistical analysis was performed using GraphPad Prism Software 9.3.1 using the appropriate tests according to normal or non-normal data distribution. Kruskal-Wallis tests were applied in Figures 2C–E, 6B,C and Supplementary Figures S2I–L. For pair-wise comparisons we performed Dunn’s multiple comparison post-hoc Kruskal-Wallis test. Mann-Whitney *U* tests were applied in Supplementary Figures S1G,H, S2A,B. Data were shown as mean ± SD or SEM as depicted in the figure legends. Statistical significance was considered for *p ≤ 0.1, **p ≤ 0.01, ***p ≤ 0.001.

## Results

### A Microfluidic Set-Up to Elucidate the Chemokine Gradient Sensing Mechanisms of Dendritic Cells

To elucidate the chemokine gradient sensing strategy of DCs, we employed a combination of microfluidics and quantitative confocal live cell imaging, which allows to analyze the response of motile cells towards controlled chemokine gradients with high spatio-temporal resolution. In our “source-sink”-based microfluidic approach, laminar flows from three independent source channels were merged and hydro-dynamically focused to generate soluble concentration gradients of chemokines with a sigmoidal-shaped concentration profile (Figure 1A). This profile includes sections that are approximately exponential, linear, and asymptotic (Figures 1A,B, 2A). Utilizing automatic pneumatic feedback regulation (flow rate control) and executable shell script files (Figure 1A; Supplementary Table S1) enabled specific adjustment of flow rates of the three source channels and subsequent precise spatio-temporal control of the gradient’s steepness and position. This set-up allows therefore to expose cells to either soluble stable gradients (Figures 1B,C) or to dynamic chemokine environments (Figures 1D–F), i.e., abrogation of the gradient, and dynamic oscillatory stimulation (traveling waves) (Nakajima and Sawai, 2016).

10 kDa Alexa Fluor 647-dextran, which diffuses similarly to Alexa Fluor 647-CCL19, was utilized to characterize the gradient (Supplementary Figure S1A). In initial chemotaxis studies we used labeled Alexa Fluor 647 CCL19 to exclude chemotactic effects caused by dextran (data not shown). We employed microfluidics on a 2D surface (Supplementary Figure S1B), since gradients in under agarose assays (Lauffenburger et al., 1983; Heit and Kubes, 2003) and in 3D collagen scaffolds (Haessler et al., 2011; Petrie Aronin et al., 2017; Frick et al., 2018) rely on diffusion at large scales, and thus require at least 30 min for gradient stabilization. These systems are, consequently, not suitable to generate fast dynamic temporal patterns of stimulation, e.g., gradient removal or traveling waves. In contrast to under agarose and 3D collagen assays, in our 2D model the cells were not confined (Supplementary Figure S1B) which excludes stimulation of cell motility by geometrical constrains. Our flow-based 2D microfluidic assay is characterized by fast gradient stabilization, i.e., both the final gradient slope and the absolute concentration of CCL19 become stable within ∼3 min (Supplementary Figure S1C). The cells were placed into the microfluidic chamber-slide before they are exposed to the gradient. Fast *de novo* formation and stabilization of the gradient implicates that DCs sense the steady-state gradient for the first time in approximately 3 min. Due to precise feedback-control and pulseless flow, our pneumatic device generates stable gradients over a period of at least 60 min (Supplementary Figure S1D) without oscillating noise, which is detrimental when syringe pump based microfluidic approaches were used instead of pneumatic devices (Supplementary Figure S1E). The microfluidic setup combined with fast confocal live cell imaging of LPS-activated motile bone marrow-derived DCs (BM-DCs) and CD11c+ splenic DCs, which both were isolated from Lifeact-EGFP mice (Riedl et al., 2010), allowed us to analyze trajectories of motile cells, velocity, displacement, directionality, actin dynamics, and cytoskeletal polarization (Supplementary Figure S1F).

### Dendritic Cells Migrate With High Directional Persistence in Stable Soluble CCL19 Gradients

To elucidate how BM-DCs sense and integrate the information of chemotactic cues into a directional movement, we acquired trajectories of BM-DCs migrating along stable soluble CCL19 gradients over a period of 60 min and subsequently determined accumulated distance and directionality by measuring cell displacements for time intervals of 2–20 s (Figure 2). The directionality parameter is a measure of straightness of motile cells without consideration of a gradient source direction. This parameter is the linear distance between start- and endpoint of the migration tracks, divided by the accumulated distance of the tracks. The Forward Migration Index (FMI) is analogous to a chemotactic index (Parr et al., 2019; Frattolin et al., 2021) and used to compare a cell’s most direct path to the chemokine gradient source to its total accumulated distance (Supplementary Figure S1F). Since cells were exposed to CCL19 gradients which were aligned horizontally in the field of view, we used the prefix “x” (x-FMI). An index equal to one, x-FMI = 1, represents perfect migration along the direction of the gradient, and x-FMI = −1 migration against the gradient direction. We observed that BM-DCs migrate with high directional persistence (Mean Directionality = 0.63, mean x-FMI = 0.62) in a steep “CCL19 gradient” region of ∼150 µm width (Figures 2A–E; Supplementary Video S1), which resembles gradients of CCR7 ligands with steep decay detected in perilymphatic regions (Weber et al., 2013). In this “steep” CCL19 gradient region, BM-DCs reached a mean accumulated distance of 93.9 µm over a time period of 60 min (Figure 2C). In contrast, cells migrating in areas characterized by a shallow exponential decay of the gradient (“CCL19 low”) or in approximately uniform concentration of 50 nM CCL19 (“CCL19 plateau”), exhibited a significantly reduced directional persistence with a mean accumulated distance below 30 µm and a mean x-FMI below 0.06 (Figures 2C,E). We observed that in both latter regions accumulated distance and x-FMI of motile BM-DCs were similar to those cells which were tracked without CCL19- or with uniform CCL19-stimulation, respectively (Supplementary Figure S1G). We then analyzed directional migration of BM-DCs towards single standing CCL19-waves which represent a different type of stable (stationary) chemokine gradient environment. Standing waves are characterized by symmetric bell-shaped architecture, in which the concentration profiles in front and back halves of the wave are equal in strength, but opposite in direction (Figure 1C). We observed that a stable standing CCL19-wave initiates directional migration of BM-DCs from opposite direction, because the cells are attracted from both halves of the wave (Figure 3; Supplementary Video S2). The trajectories clearly show that motile BM-DCs accumulate in the peak region of the standing wave, where the absolute CCL19 concentration is highest (50 nM) and reaches asymptotic (uniform) characteristics. This chemotactic behaviour is in concordance with spatial sensing, in which cells very precisely follow the increasing concentration of two opposing gradients which are mapped out in this highly defined spatial situation. Our results clearly revealed that steady-state CCL19 gradient information is sufficient to promote directional persistent migration of DCs. We did not observe the requirement of rising absolute CCL19 concentration over time for long-range BM-DC migration towards chemokine attractants as proposed by (Petrie Aronin et al., 2017), implying that spatial sensing mechanisms control chemotaxis of BM-DCs.

### Leading Edge Protrusions and Persistent Chemotaxis of Dendritic Cells are Abrogated in the Range of Seconds When Stable Soluble CCL19 Gradients Are Perturbed

Next, we sought to determine how chemotaxing BM-DCs respond to a perturbation of chemokine gradients, since gradients *in vivo* are presumably not stable over long distances for long periods of time (Weber et al., 2013; Majumdar et al., 2014; Ulvmar et al., 2014; Sarris and Sixt, 2015; Vaahtomeri et al., 2017). Putative fluctuations of spatial gradient information raise important questions about navigation strategies of motile cells. Do chemotaxing cells abruptly stop when they are faced with an abrupt collapse of spatial gradient information, or will they instead maintain persistent motion? Although several studies investigated general chemotaxis of leukocytes (Zigmond, 1978; Friedl et al., 2001; Iijima et al., 2002; Haessler et al., 2011; Sarris and Sixt, 2015; Petrie Aronin et al., 2017; Schwarz et al., 2017; Frick et al., 2018; Renkawitz et al., 2018), directed cell behavior and stopping time of motile BM-DCs following removal of spatial cues has not been quantitatively addressed yet. To analyze how BM-DCs sense and respond to gradient perturbation *in vitro* requires high spatio-temporal control of soluble chemokines. We therefore used CCL19, which in contrast to the related chemokine CCL21, lacks the highly charged C-terminal extension that binds glycosaminoglycan (GAG) which is utilized to immobilize CCL21 to matrix proteins (Paz et al., 2007). Immobilized CCL21 therefore directs cellular haptotaxis which we avoided here, because immobilized chemokine gradients would be static and could not be manipulated in the ultra-fast and highly resolved spatio-temporal fashion which is a key innovative feature of our study. On the other hand, we provide evidence here that the efficiency of directed BM-DC migration is not significantly different, when fully soluble, stable gradients of either CCL19 or CCL21 are employed, respectively (Supplementary Figure S1H). Therefore, the functions of CCL19 and CC21 in the settings of “pure” chemotaxis investigated here are highly similar.

First of all, we recorded trajectories of motile BM-DCs over a period of 60 min, and subsequently determined x-displacement of cells towards the CCL19-source before and following gradient perturbation, i.e., removal of the soluble gradient or switch to a uniform CCL19 stimulation, respectively. In contrast to the accumulated distance, which reflects the summation of an entire path that a given cell has migrated (see above, Figure 2C), the x-displacement is here used to map the increment of cell track length over time. Note that our flow-based microfluidic approach allows for completion of chemokine gradient removal within 14 s (Figure 4A). We observed that BM-DCs were not able to maintain directional migration when CCL19 gradients were removed (Figure 4B; Supplementary Video S3) or shifted to a uniform stimulation with a final concentration of 50 nM CCL19 (Figure 4C; Supplementary Video S4). Quantification of x-displacement revealed that BM-DCs lost their directional track within ∼50 s following CCL19 gradient removal and within ∼20 s after switching from a CCL19 gradient to a uniform CCL19 stimulation, respectively.

Previously, it was shown that in fast-migrating amoeboid like cells, such as in leukocytes, actin-driven protrusions in the form of lamellipodia and filopodia facilitate directional decisions and invasion of complex matrices (Leithner et al., 2016), while actual translocation is driven by the cell body (Stanley et al., 2008). We therefore investigated whether spatial information of stable CCL19 gradients is required to maintain cytoskeletal polarization in BM-DCs. To this end, actin-driven leading-edge protrusions in Lifeact-EGFP BMDCs were visualized upon CCL19-gradient removal or re-establishment, respectively. Precise spatio-temporal positioning of soluble gradients (without significant oscillating noise), combined with fast high-resolution image acquisition (1 frame/2 s; ×63 objective), allowed us to resolve the cytoskeletal organization of chemotactic signal interpretation in response to dynamic CCL19 stimulation at the submillimeter scale. Kymograph analysis of actin-dynamics of the cell’s front (Figure 5A, yellow lines) revealed that the maintenance of leading-edge protrusions strongly depend on the presence of stable chemokine gradients, as polarized lamellipodia retracted in the range of seconds when the CCL19 gradient was removed (Figure 5B, plotted kymograph/time-space-plots of yellow lines in Figure 5A and Supplementary Video S5). This process is reversible, since BM-DCs immediately started to re-polarize when stable CCL19 gradients were re-applied (Figure 5C). Taken together, our findings revealed a requirement of stable chemokine gradient information to determine and to maintain directional persistent migration of BM-DCs by stabilizing actin-driven leading-edge protrusions. These findings strongly corroborate the concept of spatial sensing as a key navigation strategy of DCs.

### Dendritic Cells Require Spatial Gradient Information for Navigation in Dynamic CCL19 Environments

Recently, it was shown that DCs trigger local chemokine secretion from lymphatic endothelial cells (LECs), and that dynamic rather than pre-patterned immobilized chemical cues promote DC entry into initial afferent lymphatic vessels (Weber et al., 2013; Majumdar et al., 2014; Ulvmar et al., 2014; Sarris and Sixt, 2015; Vaahtomeri et al., 2017), which transport tissue fluids and leukocytes into the subcapsular sinus of draining lymph nodes. Navigation of cells in such complex and dynamic environments requires integration of spatial and temporal cues which are poorly understood. To gain insight into the functional coupling of directional sensing and decision-making, we analyzed the migratory behaviour of BM-DCs in response to fast (<10 s) 180-degree direction switching of soluble CCL19 gradients. We observed that BM-DCs instantaneously sense the altered chemokine stimulus and rectify their migration path toward the newly established CCL19 gradient (Supplementary Video S6). We conclude from these findings that BM-DCs employ a spatial directional sensing strategy to sense dynamics and changes of gradient direction to facilitate decision-making in directional navigation.

The observed capability of DCs to sense and respond to changes in spatial gradient direction raised the question of whether BM-DCs are also able to decipher dynamic oscillatory CCL19 environments. To this end, we exposed BM-DCs to traveling CCL19 waves (Figure 1F), which represent an established type of dynamic spatio-temporal stimulation (Nakajima et al., 2014; Skoge et al., 2014). Recent studies have shown that *Dictyostelium* amoeba have the capability to decipher dynamic spatio-temporal stimulation, in which they “ignore” the decreasing gradient in the back half of dynamic traveling waves, and continue to migrate with high directional persistence towards the source of cAMP (Nakajima et al., 2014; Skoge et al., 2014). We observed that, in contrast to *Dictyostelium* (Supplementary Figures S2A–C)*,* BM-DCs do not employ adaptive temporal sensing strategies that discriminate increasing and decreasing concentrations of chemoattractant (Figures 6A–E; Supplementary Figures S2D–J). In contrast to stable gradients, traveling CCL19 waves over a wide range of period length (τ = 2–30 min) did not induce persistent directional migration of DCs, since mean accumulated distances of BM-DCs are below 30 µm in 60 min and mean x-FMI are below 0.1 (Figures 6B,C). We then analyzed the x-displacement of DCs, which is here used to map the track length of cells towards the source of individual CCL19 wave pulses (τ = 2, 4, 6, 15, or 30 min). Interestingly, analysis of x-displacement in single traveling waves revealed that DCs transiently respond to the front half of the wave (increasing CCL19 concentration) when wave propagation was very slow (Figure 6D; τ = 30 min). However, these measures of length (x-displacement ≤ 15 µm) are below those of BM-DC’s cell size and therefore might not indicate real net translocation of the cell body. To gain insight into the actual locomotion process, we therefore developed a specific high-resolution kymograph approach, which allows the precise analysis of actin dynamics in response to periodic CCL19 wave stimulation. We observed that, in contrast to stable gradients, traveling CCL19 waves (τ ≥ 6 min) only induced the generation of short-term actin-driven membrane protrusions in the front half of the waves, but immediately retract in the back of the waves (Figure 6E; Supplementary Figures S2D–H). High-resolution confocal live cell imaging revealed, that BM-DCs responded to the oscillating stimulus with rotating actin dynamics around the entire cell periphery (Supplementary Video S7) instead of a cytoskeletal polarization (leading edge formation) towards the CCL19 wave source. We hypothesize from these results, that BM-DCs in fact sensed the CCL19 signal when they were exposed to slow waves (τ = 15–30 min; Figure 6E; Supplementary Figures S2D–H), but that the cells do not induce directional migration when the CCL19 gradient fluctuates. Obviously, the recurrent decreasing concentration gradients in the back halves of the waves prevent the formation of stable leading edge lamellipodia and subsequent directional decision-making of BM-DCs, when exposed to oscillating CCL19 stimulation. These results strongly corroborate our concept (based on Figures 2, 3) that DCs recognize a differential in chemokine concentration across their cell diameter *via* a mechanism referred to as “*spatial recognition*” (Berg and Purcell, 1977), since directionality is immediately abrogated when cells experience gradient stagnation or removal (also see above, Figure 4). To exclude that the observed spatial sensing behavior of BM-DCs toward CCL19 gradients is merely based on specific CCR7 receptor signaling, we performed additional experiments with other chemotactic stimuli, which are known to play significant roles in DC trafficking (Shin et al., 2006; Del Prete et al., 2007; Chen et al., 2013). Our results provide evidence that the spatial sensing strategy of BM-DCs is based on cell intrinsic regulation and not on specific CCR7 receptor signaling, since BM-DCs show similar spatial sensing behavior when exposed to ligands of other G protein-coupled receptors, like CXCR4, BLT1/2 or FPR2/3, respectively (Supplementary Figures S2I,J).

In essence, the observed capability of BM-DCs to migrate with high directional persistence in steady-state gradients but not in traveling CCL19 waves is compatible with a gradient sensing strategy in which BM-DCs require spatial gradient information for efficient chemotaxis. In contrast to *Dictyostelium* (Nakajima et al., 2014; Skoge et al., 2014), BM-DCs do not employ adaptive temporal sensing to decipher periodic-temporal stimulation (Figure 6A). These results were strongly confirmed by results obtained with primary CD11c+ splenic DCs (Supplementary Figures S2K–M) displaying a migratory behaviour which is in concordance with a spatial navigation strategy which we observed in BM-DCs.

Taken together our analysis revealed that persistent chemotaxis of DCs depends on continuously present spatially organized cues which they sense on the sub-millimeter scale, and we furthermore showed that these cells do not require temporally increasing stimulation by chemoattractants for directed motion. The observed capability of DCs to migrate with high directional persistence in stable gradients but not in periodic temporal cues identifies spatial sensing as a key requirement for persistent chemotaxis of DCs.

## Discussion

In the present study, we employ chemokine-gradient-guided migration to elucidate the sensing mechanism of motile DCs. To this end, we set up a high-resolution microfluidic device to expose DCs to precisely controlled, temporally and spatially varying soluble CCL19 gradients, while simultaneously tracking actin dynamics, cell polarization, and chemotaxis. Our data demonstrate that efficient guidance of DCs depends on the translation of chemokine-encoded spatial information of the gradient into directionally persistent migration. We observed that steady-state CCL19 gradients are sufficient for chemotactic sensing of DCs, without the requirement of integrating a temporal evolution of increasing concentration signals. Our experiments furthermore show that DCs do not employ adaptive temporal sensing strategies in our setting, since cells are not able to maintain cell polarity and directional persistence when they are exposed to oscillating chemotactic cues.

Our observations support a concept of spatial sensing in which DCs interpret the concentration difference of chemokines integrated by the difference of receptor occupancy at different positions in the cell, a theory made in many models of signal recognition in spatial sensing and directional migration (Bagorda and Parent, 2008; Lauffenburger et al., 1983; Parent and Devreotes, 1999; Schwarz et al., 2017). Our data clearly show, that DCs have the capability to integrate spatial chemokine information and to migrate with high directional persistence toward steady-state soluble CCL19 gradients (>60 min) on 2D fibronectin (Figures 2, 5). This is in line with previous studies demonstrating similar persistent migratory responses of motile DCs toward stable chemokine gradients in a 3D environment (Haessler et al., 2011; Frick et al., 2018; Lämmermann and Sixt, 2009; Quast et al., 2011; Schumann et al., 2010). We do not observe a requirement for increasing absolute stimulus levels over time, in contrast to a recent study which proposed that temporal sensing mechanisms control prolonged chemotaxis of myeloid cells (Petrie Aronin et al., 2017), but we of course concede that we did not precisely replicate the experimental conditions of that particular study. For example, as opposed to (Petrie Aronin et al., 2017), we did not employ a 3D-system which would prohibit ultra-fast manipulation of the gradients. It is indisputable that 3D cellular *in vitro* environments are in comparison to 2D cell culture settings more akin to the natural environment of DCs found in tissues (Randolph et al., 2005; Worbs et al., 2017). Furthermore, it is established that some aspects of cell migration differ on 2D substrates as compared to migration in 3D environments, e.g., leukocytes are able to migrate in the absence of adhesion receptors within 3D interstitial space (Lämmermann and Sixt, 2009). However, in various diffusion-based microfluidic devices which have been developed to investigate the migration behavior of DCs in 3D collagen environments, both slow *de novo* development of the gradient (30–120 min) as well as slow response time for dynamic modulation of the gradient (Haessler et al., 2011; Petrie Aronin et al., 2017; Frick et al., 2018) are detrimental when it is aimed to analyze sensing strategies of DCs with high spatio-temporal resolution. For this reason we performed precise chemotaxis assays in flow-based source-sink microfluidics on 2D substrates analogous to the approach recently described for motility assays with *Dictyostelium* amoeba (Nakajima et al., 2014). Our flow-based microfluidic approach is characterized by fast stabilization of the final gradient slope as well as the possibility to apply fast modeling of dynamic stimulatory conditions with high spatio-temporal precision (e.g., gradient removal or traveling waves, respectively).

Although we clearly observed that DCs are able to respond to spatial chemokine gradient information with persistent directional migration *in vitro*, it is inconceivable that steady-state spatial gradients are stable over long distances for long periods of time *in vivo* (Lämmermann et al., 2013; Cai and Montell, 2014; Sarris and Sixt, 2015; Ulvmar et al., 2014). Physiological chemokine gradients are presumably instable as a result of hindered chemokine diffusion (Ulvmar et al., 2014), chemokine immobilization (Sarris and Sixt, 2015), or dynamic chemokine release (Wessels et al., 1992). Recently, it has been shown that DCs do not encounter pre-immobilized CCR7-ligands but trigger local CCL21 release from LECs and that dynamic rather than pre-patterned immobilized chemical cues promote DC entry into afferent lymphatics (Vaahtomeri et al., 2017). The presence of dynamic chemical gradients would require an integration of spatial and temporal information which is poorly understood. Migration principles established for rapid amoeboid crawling leukocytes share many characteristics with those described for the social amoeba *Dictyostelium* (Friedl et al., 2001). It is generally accepted that both cell types employ spatial sensing in static chemokine gradients to induce actin-driven cell polarity and directional migration (Parent and Devreotes, 1999; Iijima et al., 2002), but if these cell types are able to maintain directional migration when the guidance cue is oscillating remained elusive. Recent studies have shown that cellular memory and adaptive temporal sensing, which combines the integration of spatial and temporal information, enables *Dictyostelium* to migrate toward traveling waves of cAMP (Nakajima et al., 2014; Skoge et al., 2014). Since leukocytes share many characteristics of so-called amoeboid crawling movement described for *Dictyostelium* (Friedl et al., 2001; Lämmermann and Sixt, 2009; Ishikava-Ankerhold et al., 2022) we utilized the setup of oscillating traveling waves (Figure 6), which represents an established surrogate model of dynamic spatio-temporal stimulation (Nakajima et al., 2014; Skoge et al., 2014). Measurements of cell displacement towards the wave source revealed substantial differences in chemotactic sensing between *Dictyostelium* and DCs. We observed that DCs were not ignoring decreasing chemoattractant concentration in the back halves of CCL19 traveling waves. Therefore, and in contrast to amoeba, DCs apparently do not employ adaptive temporal sensing strategies that discriminate temporally increasing and decreasing chemoattractant concentrations in our setting. Previous studies which have shown that DCs interpret haptotactic immobilized chemokine gradients strongly support the hypothesis that DCs employ spatial sensing (Schumann et al., 2010; Schwarz et al., 2017). However, that integration of soluble chemokine gradient information is solely based on spatial sensing strategies of DCs is, to our knowledge, first evidenced by the present study. Our observation that cells depolarize and stop migrating instantaneously following CCL19 gradient abrogation strongly corroborates the notion that spatial gradient information is essential to maintain directional DC migration. However, DCs have the ability to instantly sense the reversed direction of a stable gradient, since we observed that these cells rectify their direction within seconds following CCL19 gradient switch (Supplementary Video S6).

## Data Availability

The original contributions presented in the study are included in the article/Supplementary Material, further inquiries can be directed to the corresponding author.

## Supplementary Material

Supplementary Table S1 The table summarizes flow rates, corresponding gradient shape of the chemoattractant, and the spatio-temporal condition of the gradient (stable, dynamic) which were used in respective experiments.

Supplementary Figure S1
**(A)** Sigmoidal shaped concentration profile of Alexa 647 Fluor recombinant (rec) CCL19 compared to the profile of the surrogate molecule, 10 kDa Alexa 647 Fluor dextran. **(B)** Graphical representation of the microfluidic chamber (lateral view). **(C)** Concentration profiles (gradient levels) of 10 kDa Alexa 647 Fluor dextran during de novo formation of the gradient. **(D)** Concentration profiles of 10 kDa Alexa 647 Fluor dextran (gradient levels) 30–60 min after establishment of stable gradients. **(E)** Comparison measurement of gradient stability. Concentration profiles of 10 kDa Alexa 647 Fluor dextran, measured in 10 s time intervals 30 min after establishment of stable gradients which are either generated by pneumatic or syringe pumps. **(F)** Graphical representation of migration parameter analysis following live cell imaging and cell tracking. FMI (=Forward Migration Index) is analogous to a Chemotaxis Index and used to compare a cell’s most direct part to the chemokine gradient. **(G)** Analysis of BM-DCs migration for 60 min without CCL19 or exposed to uniform CCL19 stimulation (50 nM), including accumulated distance, directionality and x-FMI. Representative data from one of 3 experiments; triangles represent randomly chosen cells per area, *N* = 15–18, mean ± SD. Statistical tests: ns, non-significant, Mann-Whitney *U* test. **(H)** Analysis of BM-DCs migration for 60 min towards a CCL19 or a CCL21 gradient, respectively. The analysis includes accumulated distance, directionality and x-FMI. Representative data from one of 3 experiments; triangles represent randomly chosen cells per area, *N* = 15–18, mean ± SD. Statistical tests: ns, non-significant, Mann-Whitney *U* test.

Supplementary Figure S2
**(A,B)** Migration analysis of Dictyostelium discoideum strain AX2 (clone: EB27-3-4, expressing LimEdeltacc-GFP) for 60 min exposed to stable soluble cAMP gradients or cAMP traveling waves (τ = 6 min), including **(A)** accumulated distance and **(B)** x-Forward Migration Index (x-FMI) of individual cells. Each bar is representative of 4 experiments with randomly chosen cells per condition, *N* = 12, mean ± SEM. **(C)** X-displacements of AX2 cells for 60 min towards the source of soluble traveling cAMP waves (τ = 6 min). Representative data from one of 4 experiments with randomly chosen cells per condition, *N* = 12, mean ± SEM. **(D–H)** High resolution kymographic analysis of actin dynamics of Lifeact-EGFP expressing BM-DCs which are exposed to traveling CCL19 waves with various periods: **(D)** τ = 2 min, **(E)** τ = 6 min, **(F)** τ = 15 min, **(G)** τ = 30 min, or **(H)** soluble stable CCL19 gradients, respectively. Actin dynamics of individual cells were analyzed by kymograph and line scan analysis of yellow lines. Representative data from one of 3 experiments for each condition, *N* = 3–12. White star in **(H)** indicates persistent displacement of the leading edge (EGFP-Lifeact signal), indicating directional net cell translocation. **(I,J)** Analysis of BM-DC migration for 60 min exposed to stable soluble gradients or traveling waves (τ = 6 min) of various chemotactic cues (CXCL12, LTB4, FPR2/3 receptor agonist WKYMVM), including **(J)** x-displacement and **(K)** x-Forward Migration Index (x-FMI) of individual cells. Representative data from one of 3 experiments; triangles represent randomly chosen cells per condition, *N* = 13–16, mean ± SD. **(K,L)** Migration analysis of primary CD11c+ splenic DCs for 60 min exposed to stable soluble CCL19 gradients or traveling CCL19 waves with various periods (τ = 2 min, 6 min), including **(L)** accumulated distance and **(M)** x-Forward Migration Index (x-FMI) of individual cells. Representative data from one of 3 experiments; triangles represent randomly chosen cells per condition, *N* = 12, mean ± SD. **(M)** X-displacements of primary CD11c+ splenic DCs for 60 min towards the source of soluble traveling CCL19 waves with various periods (τ = 2 min, 6 min). Representative data from one of 3 experiments with randomly chosen cells per condition, *N* = 12, mean ± SD. **(D–H)** Green = Lifeact-EGFP expressing BM-DCs, red = 10 kDa Dextran AF 647. Statistical tests: **(A,B,I–L)** ****p* ≤ 0.001, ***p* ≤ 0.1, ns, non-significant, **(A,B)** Mann-Whitney *U* test, **(I–L)** Dunn’s multiple comparison (post-hoc Kruskal-Wallis test).

Supplementary Video S1 Chemotaxis of Lifeact-EGFP expressing BM-DCs (green) towards a stable soluble CCL19 gradient.The gradient is visualized with the help of the surrogate molecule 10 kDa Dextran Alexa Fluor 647 (red). Images were acquired every 2 s. Period of time, 61 min.

Supplementary Video S2 Analysis of BM-DCs migration towards both halves of a symmetric bell-shaped standing CCL19 wave. Tracks of individual motile Lifeact-EGFP expressing BM-DCs (green) are pseudo-colored (time is color-coded: blue = 0 min, red = 60 min). The gradient is visualized with the help of the surrogate molecule 10 kDa Dextran Alexa Fluor 647 (red). Images were acquired every 2 s. Period of time, 60 min.

Supplementary Video S3 Directional persistent migration of BM-DCs is abrogated in the range of seconds when a stable CCL19 gradient is perturbed. Chemotaxis of Lifeact-EGFP expressing BM-DCs (green) towards a stable soluble CCL19 gradient and following CCL19 gradient removal. Instant time of gradient removal: 30 min following start of the video. The gradient is visualized with the help of the surrogate molecule 10 kDa Dextran Alexa Fluor 647 (red). Colored lines mark trajectories of motile cells following manual tracking. Images were acquired every 2 s. Period of time, 52 min.

Supplementary Video S4 Directional persistent migration of BM-DCs is abrogated in the range of seconds when a stable CCL19 gradient is perturbed. Chemotaxis of Lifeact-EGFP expressing BM-DCs (green) towards a stable soluble CCL19 gradient and following uniform CCL19 stimulation. Instant time of gradient abrogation: 46 min following start of the video. The gradient is visualized with the help of the surrogate molecule 10 kDa Dextran Alexa Fluor 647 (red). Colored lines mark trajectories of motile cells following manual tracking. Images were acquired every 2 s. Period of time, 56 min.

Supplementary Video S5 Steady-state chemokine gradients are essential to stabilize leading edge protrusions in BM-DCs. Actin-dynamics and leading-edge polarization of a Lifeact-EGFP expressing BM-DC (gray) in a stable soluble CCL19 gradient and subsequent depolarization of the cell in response to CCL19 gradient removal. Instant time of gradient removal: 2.5 min following start of the video. Images were acquired every 2 s. Period of time, 5 min.

Supplementary Video S6 Chemotaxing BM-DCs sense and response to 180-degree direction switching of soluble CCL19 gradients and instantaneously rectify their migration path towards the new established CCL19 gradient. Colored tracks mark trajectories of Lifeact-EGFP expressing BM-DCs (green) towards stable soluble CCL19 tracks following manual tracking (left panel). Corresponding cell trajectories are plotted on the right panel. The gradient is visualized with the help of the surrogate molecule 10 kDa Dextran Alexa Fluor 647 (red). Images were acquired every 2 s. Period of time, 85 min.

Supplementary Video S7 Behavior of Lifeact-EGFP expressing BM-DCs (green) exposed to dynamic traveling CCL19 waves (τ = 6 min). The bell-shaped waves are visualized with the help of the surrogate molecule 10 kDa Dextran Alexa Fluor 647 (red). Images were acquired every 2 s. Period of time, 50 min.
