# Supplementary figures and images for: A Stable Chemokine Gradient Controls Directional Persistence of Migrating Dendritic Cells
Source: Front Cell Dev Biol. 2022 Aug 9;10:943041. doi: 10.3389/fcell.2022.943041 (PMC9395945; doi:10.3389/fcell.2022.943041)

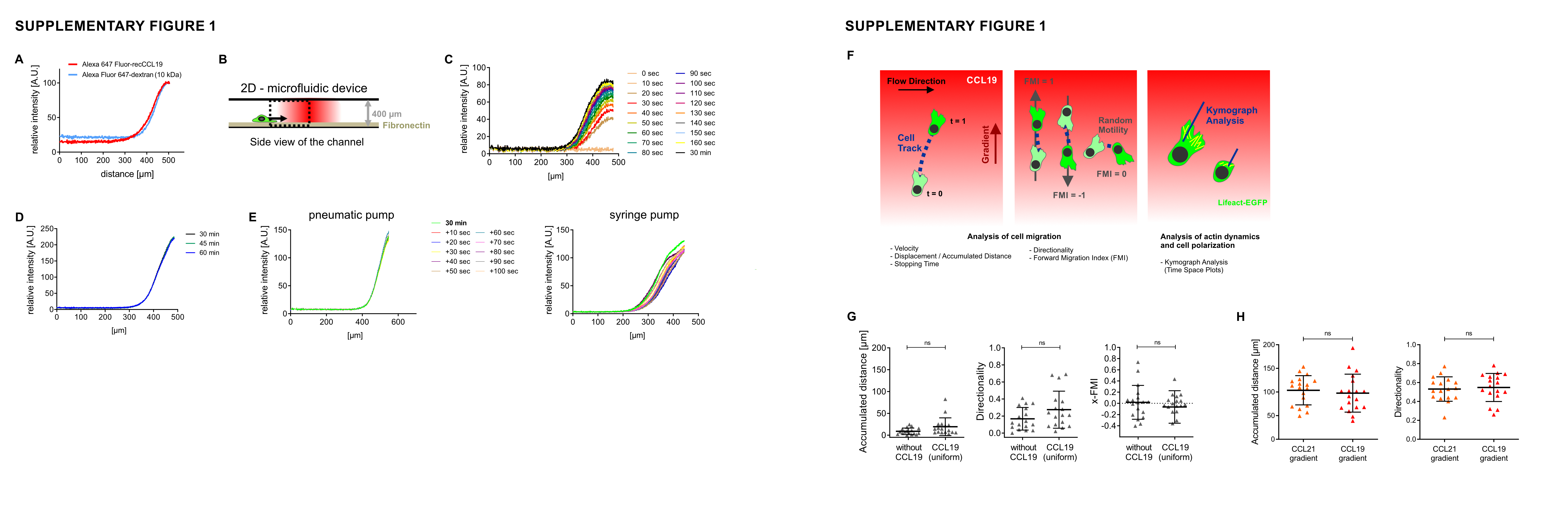

Supplement: Supplementary file 1 [file Image1.tiff]

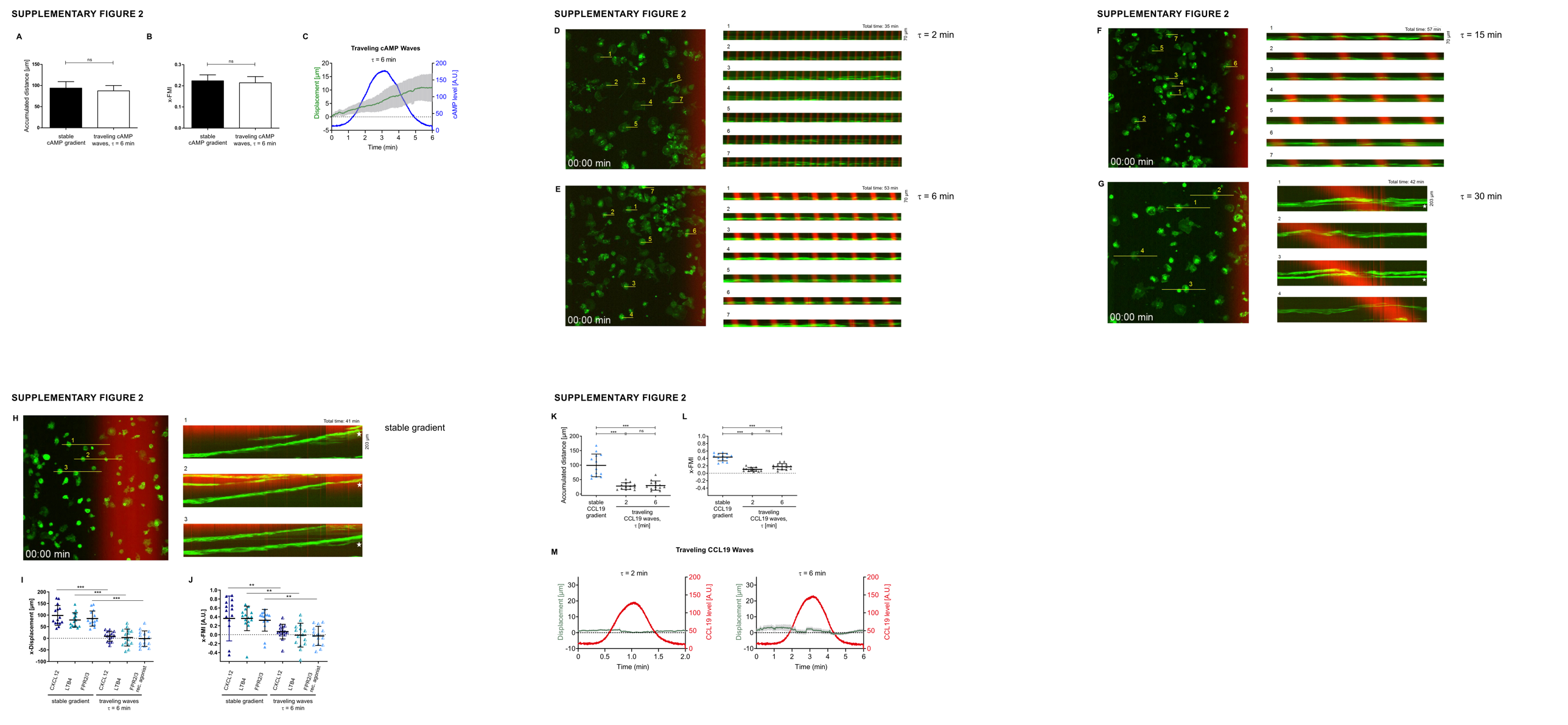

Supplement: Supplementary file 7 [file Image2.tiff]
