# Supplementary material for: A Stable Chemokine Gradient Controls Directional Persistence of Migrating Dendritic Cells
Source: Front Cell Dev Biol. 2022 Aug 9;10:943041. doi: 10.3389/fcell.2022.943041 (PMC9395945; doi:10.3389/fcell.2022.943041)
Supplement: Supplementary file 3 [file Table1.pdf]

SUPPLEMENTARY TABLE 1

Figure	Flow rate (µl/min) Configuration<br>Maesflow software (Fluigent)			Condition	Comment /<br>Reference
	Upper Inlet	Central Inlet	Lower Inlet		
**2**	70	10	70	Stable gradient (60 min)	
**3**	70	10	70	Standing wave (t = 60 min),	
**4A**	70<br>0	10<br>0	70<br>70	Stable gradient (t = 0 s),<br>Gradient removal (t ≥ 2 s)	
**4B**	70<br>0	10<br>0	70<br>70	Stable gradient (t = 0 min),<br>Gradient removal (t ≥ 30 min)	
**4C**	70<br>0	10<br>70	70<br>0	Stable gradient (t = 0 s),<br>Uniform CCL19 stimulation (t ≥ 45 min)	
**5A, B**	70<br>0	10<br>0	70<br>70	Stable gradient (t = 0 – 2.5 min),<br>Gradient removal (t = 2.5 – 5.0 min)	
**5C**	70<br>0	10<br>0	70<br>70	Stable gradient (t = 0 – 2.5 min),<br>Gradient removal (t = 2.5 – 5.0 min)<br>**Continuous repetition over a period of 30 min**	
**6**	70<br><br><br>70<br>70<br>70<br><br>70<br>70<br>70<br><br>70<br>70<br>70<br><br>70<br>70<br>70	10<br><br><br>10<br>10<br>10<br><br>10<br>10<br>10<br><br>10<br>10<br>10<br><br>10<br>10<br>10	70<br><br><br>70<br>0<br>70<br><br>70<br>0<br>70<br><br>70<br>0<br>70<br><br>70<br>0<br>70	Stable gradient (41-60 min)<br><u>Traveling waves</u><br>2 min/L_p (41-60 min)<br>(Lower insert: → decrease of flow rate: -2µl/660ms for 2 min;<br>After 2 min: increase to 70 µl/min<br><br>6 min/L_p (53 min)<br>(Lower insert: → decrease of flow rate: -2µl/2sec for 6 min;<br>After 6 min: increase to 70 µl/min<br><br>15 min/L_p (57-60 min)<br>(Lower insert: → decrease of flow rate: -2µl/5sec for 15 min;<br>After 15 min: increase to 70 µl/min<br><br>30 min/L_p (~60 min)<br>(Lower insert: → decrease of flow rate: -2µl/10sec for 30 min;<br>After 30 min: increase to 70 µl/min	Modified executable MFCS shell script file named “travelingwave.sh.” from (Nakajima and Sawai, 2016)
**S1A, D, E** (pneum.)	70	10	70	Stable gradient (30-60 min)	
**S1G** (left scatterplot)	70	10	70	Stable gradient (60 min), without CCL19	
**S1G** (right scatterplot)	10	70	10	Uniform CCL19 stimulation (60 min)	
**S1H**	70	10	70	Stable gradient (60 min)	
**S2A-B** (left bars)	70	10	70	Stable gradient (60 min)	

**S2A-B** (right bars), **C**	70<br>70<br>70	10<br>10<br>10	70<br>0<br>70	<u>Traveling waves</u><br>6 min/L_p (53-60 min)<br>(Lower insert: → decrease of flow rate: -2µl/2sec for 6 min;<br>After 6 min: increase to 70 µl/min	Modified executable MFCS shell script file named “travelingwave.sh.” from (Nakajima and Sawai, 2016)
**S2D-H**	70<br><br>70<br>70<br>70<br><br>70<br>70<br>70<br><br>70<br>70<br>70<br><br>70<br>70<br>70	10<br><br>10<br>10<br>10<br><br>10<br>10<br>10<br><br>10<br>10<br>10<br><br>10<br>10<br>10	70<br><br>70<br>0<br>70<br><br>70<br>0<br>70<br><br>70<br>0<br>70<br><br>70<br>0<br>70	Stable gradient (41-60 min)<br><u>Traveling waves</u><br>2 min/L_p (41-60 min)<br>(Lower insert: → decrease of flow rate: -2µl/660ms for 2 min;<br>After 2 min: increase to 70 µl/min<br><br>6 min/L_p (53 min)<br>(Lower insert: → decrease of flow rate: -2µl/2sec for 6 min;<br>After 6 min: increase to 70 µl/min<br><br>15 min/L_p (57-60 min)<br>(Lower insert: → decrease of flow rate: -2µl/5sec for 15 min;<br>After 15 min: increase to 70 µl/min<br><br>30 min/L_p (~60 min)<br>(Lower insert: → decrease of flow rate: -2µl/10sec for 30 min;<br>After 30 min: increase to 70 µl/min	Modified executable MFCS shell script file named “travelingwave.sh.” from (Nakajima and Sawai, 2016)
**S2I, J**	70<br><br>70<br>70<br>70	10<br><br>10<br>10<br>10	70<br><br>70<br>0<br>70	Stable gradient (60 min)<br><u>Traveling waves</u><br>6 min/L_p (53 min)<br>(Lower insert: → decrease of flow rate: -2µl/2sec for 6 min;<br>After 6 min: increase to 70 µl/min	Modified executable MFCS shell script file named “travelingwave.sh.” from (Nakajima and Sawai, 2016)
**S2K, L** (left scatter plots)	70	10	70	Stable gradient (60 min)	
**S2K, L** (right scatter plots), **M**	70<br>70<br>70<br><br>70<br>70<br>70	10<br>10<br>10<br><br>10<br>10<br>10	70<br>0<br>70<br><br>70<br>0<br>70	<u>Traveling waves</u><br>2 min/L_p (41-60 min)<br>(Lower insert: → decrease of flow rate: -2µl/660ms for 2 min;<br>After 2 min: increase to 70 µl/min<br><br>6 min/L_p (53 min)<br>(Lower insert: → decrease of flow rate: -2µl/2sec for 6 min;<br>After 6 min: increase to 70 µl/min	Modified executable MFCS shell script file named “travelingwave.sh.” from (Nakajima and Sawai, 2016)
